# IL-1/IL-1R Signaling in Head and Neck Cancer

**DOI:** 10.3389/froh.2021.722676

**Published:** 2021-08-26

**Authors:** Sven E. Niklander, Craig Murdoch, Keith D. Hunter

**Affiliations:** ^1^Unidad de Patología y Medicina Oral, Facultad de Odontologia, Universidad Andres Bello, Viña del Mar, Chile; ^2^Unit of Oral and Maxillofacial Medicine, Pathology and Surgery, School of Clinical Dentistry, University of Sheffield, Sheffield, United Kingdom; ^3^Oral Biology and Pathology, University of Pretoria, Pretoria, South Africa

**Keywords:** head and neck cancer, squamous cell carcinoma, oral cancer, tumor microenvironment, IL-1, Anakinra (PubChem CID: 90470007)

## Abstract

Decades ago, the study of cancer biology was mainly focused on the tumor itself, paying little attention to the tumor microenvironment (TME). Currently, it is well recognized that the TME plays a vital role in cancer development and progression, with emerging treatment strategies focusing on different components of the TME, including tumoral cells, blood vessels, fibroblasts, senescent cells, inflammatory cells, inflammatory factors, among others. There is a well-accepted relationship between chronic inflammation and cancer development. Interleukin-1 (IL-1), a potent pro-inflammatory cytokine commonly found at tumor sites, is considered one of the most important inflammatory factors in cancer, and has been related with carcinogenesis, tumor growth and metastasis. Increasing evidence has linked development of head and neck squamous cell carcinoma (HNSCC) with chronic inflammation, and particularly, with IL-1 signaling. This review focuses on the most important members of the IL-1 family, with emphasis on how their aberrant expression can promote HNSCC development and metastasis, highlighting possible clinical applications.

## Introduction

The association between chronic inflammation and cancer has been reported for many years. Fifteen percent of all cancers are attributed to inflammation [1], with a well-recognized association in lung, pancreatic, esophageal, bladder, gastric, cervical, colorectal and prostate cancers [2]. Pro-inflammatory cytokines present in the tumor microenvironment (TME) can have dual effects; they can stimulate inflammation to decrease tumor progression; or they can stimulate inflammation favoring carcinogenesis, tumor growth and metastasis [3]. Cytokines are produced by host cells in response to factors secreted by the tumor cells or by the tumor itself [4, 5]. Interleukin-1 (IL-1) is commonly found at tumor sites and is considered one of the most important cytokines of the TME, where it plays a key role in carcinogenesis and tumor progression [6], and its expression has been associated with poor prognosis in cancer patients [7]. There is a growing association linking head and neck squamous cell carcinoma (HNSCC) with chronic inflammation [8, 9], in which IL-1/IL-1R signaling seems to be a key player [10]. Cumulating evidence suggests that the effects of IL-1 autocrine and paracrine signaling within the TME is central to HNSCC development. This signaling axis not only leads to increased expression of proteases and factors that dramatically alter the extracellular matrix, aiding tumor cell invasion and metastasis [2, 3], but also increases the production of leukocyte chemoattractants [11]. These molecules selectively recruit both innate and adaptive immune cells to the TME that have both anti- and pro-tumorigenic properties. Innate immune cells, such as macrophages and neutrophils, are recruited in large numbers to the HNSCC TME where they secrete tumor promoting and pro-angiogenic factors that exacerbate inflammation, and increase the supply of nutrients and oxygen that drives tumor progression. It is no surprise that high numbers of macrophages and neutrophils in the TME are associated with poor prognosis in HNSCC patients [12, 13]. In contrast, and particularly for HPV-positive HSNCC, increased numbers of T lymphocytes have been observed and these have been associated with improved prognosis due to direct anti-tumor cell targeting by these cells [14].

Here, we review the most important members of the IL-1 family, with emphasis on how their aberrant expression can promote HNSCC development and metastasis, highlighting possible clinical applications.

## IL-1 Family Members

The IL-1 family consists of several different ligands and receptors (Table 1) [16]. The most studied ligands are IL-1α and IL-1β, commonly known collectively as IL-1, and the interleukin-1 receptor antagonist (IL-1RA), which antagonizes the effects of IL-1α and IL-1β [17]. These ligands bind to IL-1 receptor 1 (IL-1R1) and IL-1 receptor 2 (IL-1R2) that are expressed by several cells. IL-1R1 is a biologically active receptor with the ability to bind to either form of IL-1 [18], while IL-1R2 is non-biologically active and acts as a decoy receptor, inhibiting the effects of IL-1 [19, 20].

**Table 1:** IL-1 family of ligands and receptors (adapted from Dinarello, [15]).

**IL-1 family member**	**Receptor**	**Function**
IL-1α	IL-1R1/IL-1R2	PI/AI
IL-1β	IL-1R1/IL-1R2	PI/AI
IL-1RA	IL1-R1	AI
IL-18	IL-1R5	PI
IL-33	IL-1R4	PI
IL-36*α, β, γ*	IL-1R6	PI
IL-36RA	IL-1R6	AI
IL-37	IL-1R5	AI
IL-38	IL-1R6	AI

### IL-1α

IL-1α is produced initially as a 31–33 kDa precursor protein (preIL-1α) that is cleaved into its 17 kDa mature C-terminal component (mIL-1 α) and a 16 kDa N-terminal propiece (ppIL-1α) by the calcium-activated cysteine protease, calpain [5, 21–23]. All forms of IL-1α are biologically active [24]. PreIL-1α lacks a leader peptide and therefore cannot be secreted and remains intracellular [17, 25]. Despite not being secreted, preIL-1α can localize on the cell surface of macrophages, endothelial cells, fibroblasts and dendritic cells [26] where is referred to as membrane-bound IL-1α. Here it acts in a juxtracrine manner by activating the IL-1R1 receptor of surrounding cells [17]. Both preIL-1α and mIL-1α are expressed constitutively in epithelial and endothelial cells and are considered to act in an autocrine or paracrine manner [27–29]. IL-1α plays an important role in inflammation acting in a juxtracrine manner [17] and has been related with several other cellular functions, such as onset of senescence [30–32], cell growth, cell differentiation [28, 33], immune response [34] and regulation of gene expression [35–37].

To exert its biological function, mIL-1α binds to IL-1R1 to trigger different cellular functions, but preIL-1α and ppIL-1α can also interact directly with the DNA without binding to IL-1R1 in a variety of cells [28, 38]. This is because ppIL-1α contains a canonical nuclear localization sequence (NLS) that enables it to interact directly within the nucleus in a non-IL-1R1-dependent manner [39, 40]. PreIL-1α, *via* ppIL-1α, interacts with histone acetyltransferases Gcn5, p300, PCAF and with the adaptor component Ada3, inducing protein transcription without activating IL-1R1 [41], and in doing so exerts different intracellular functions, such as NF-κB and AP-1 activation [38], modulation of endothelial proliferation [28], migration [42] and cytokine production [43].

### IL-1β

IL-1β is the classic inflammatory secreted cytokine produced in response to inflammatory signals and other stimuli and can act in a paracrine or systemic manner [44, 45]. IL-1β is mainly produced by monocytes by intracellular cleavage from its 31 kDa precursor protein (pIL-1β) into a 17.5 kDa mature form (mIL-1β) by caspase-1 or IL-1β converting enzyme (ICE) [5, 46]. The precursor form of IL-1β is considered to be an inactive immature form of the protein [18]. Unlike preIL-1α, pIL-1β is not expressed in health [47]. Many microbial products are able to stimulate IL-1β secretion, and when produced, IL-1β, together with IL-1α, has the ability to upregulate its own gene expression *in vitro* and *in vivo* [48]. IL-1β expression is mainly restricted to inflammatory cells, where it is regulated in response to external stimuli [49]. IL-1β is 25–50-fold more abundant than IL-1α in stimulated human peripheral mononuclear cells [50], and upon activation, around 70% of IL-1β is secreted by these cells after 24 h stimulation [51].

The main functions of IL-1β are to induce upregulation of cytokines, chemokines, adhesion molecules, acute phase proteins and tissue remodeling enzymes [17, 49, 52]; it may also act as an angiogenic factor in tumors [53], inducing the production of vascular endothelial growth factor (VEGF) *via* cyclooxygenase (COX)-2 activation [54]. IL-1β has been associated with the pathobiology of many diseases, such as familial periodic fever syndromes [55], multiple organ failure in sepsis [56], rheumatoid arthritis, type II diabetes [57], chronic obstructive pulmonary disease [58] and growth, vascularization and metastasis of malignant tumors [53].

### IL-1 Receptors

#### IL-1R1

IL-1R1 is the main receptor through which IL-1 exerts its effects and is found on T cells, keratinocytes, fibroblasts, synovial cells, endothelial cells, chondrocytes, and hepatocytes [17, 51]. All active forms of IL-1 (pIL-1α, mIL-1α and mature IL-1β) bind with similar affinity to IL-1R1, triggering biological actions [49, 59]. IL-1R1 is an 80 kDa molecular weight protein and belongs to the immunoglobulin (Ig) super family. It has a single transmembrane portion with a cytosolic region and an extracellular segment that contains three domains homologous to Igs, with seven N-linked glycosylation sites. To be active, the IL-1R1/IL-1 extracellular complex requires the additional binding of a co-receptor, the IL-1 receptor accessory protein (IL-1RAcP) or IL-1R3, forming a trimeric complex [60, 61]. IL-1RAcP is essential for signal transduction, as murine fibroblasts deficient in IL-1RAcP showed no response after IL-1 stimulation [62]. After IL-1 binds to the extracellular domain, the Toll/interleukin-1 receptor (TIR) domain of the cytoplasmic portion of IL-1R1 triggers a cascade of intracellular signaling events that result in the phosphorylation and degradation of inhibitor nuclear factor κB (IκB) [63]. This releases p50 and p65 NF-κB sub-units that upon phosphorylation are transported into the nucleus and bind to specific DNA promotor sequences, initiating gene transcription [49, 64]. Although it is generally accepted that IL-1R1 is localized on the cell membrane, recent reports have shown that the receptor is also present on the nuclear membrane of malignant oral keratinocytes [65, 66], although the relevance of this nuclear localization is unknown.

#### IL-1R2

IL-1R2 is an inactive IL-1 receptor that acts as a molecular trap, capturing IL-1 on the plasma membrane or within the extracellular space when membrane IL-1R2 is cleaved and present as a soluble receptor, without triggering agonist activity [61, 67]. Together with IL-1RA, these IL-1R2 forms act as IL-1 inhibitors [68]. IL-1R2 is also a member of the Ig super family, consisting of three Ig-like domains in the extracellular portion and a transmembrane segment. It is found on B and T helper 2 cells, neutrophils, monocytes, bone marrow and microglial cells [15]. The main difference between the two IL-1R forms is that, unlike Il-1R1, IL-1R2 has no TIR domain, and is therefore unable to trigger intracellular signaling, rendering it biologically inactive [69].

### IL-1RA

IL-1RA blocks the binding of IL-1α and IL-1β to IL-1R1, having no cross-reactivity with IL-1α and IL-1β [70]. When binding to IL-1R1, IL-1RA does not recruit IL-1RAcP. Thus, IL-1RA has no agonist action, acting as a pure antagonist molecule [71]. IL-1RA mainly binds IL-1R1, having little effect on IL-1R2, which is in agreement with their action as IL-1 inhibitor molecules [72]. As IL-1RA competes with IL-1 for the same receptor, it is found in higher concentrations than IL-1. For example, in the skin, IL-1RA expression has been found to be ≈100-fold higher than IL-1α [27]. Similarly, a study with recombinant IL-1RA showed that in order to have 50% inhibition of IL-1-induced actions, IL-1RA had to be present in 5–100-fold excess over both IL-1α and IL-1β [73]. This is because IL-1R1 is very sensitive to small amounts of IL-1. Even 5% IL-1 receptor occupancy is able to trigger a complete biological response [63]. So, for IL-1RA to efficiently block the effects of IL-1, it must be in abundance [15].

Two main forms of IL-1RA are now recognized; a secreted form (sIL-1RA) [74] and an intracellular form (icIL-1RA) [75]. Intracellular IL-1RA has the same amino acid structure as the secreted form, but lacks a leader peptide that prevents its secretion. The intracellular variant is transcribed by alternate splicing of the same gene as sIL-1RA [75]. Three isoforms of the intracellular form have been reported (icIL-1RA1 or transcript variant 3, icIL-1RA2 or transcript variant 2 and icIL-1RA3 or transcript variant 4) [76–78], with icIL-1RA1 being the most studied. Both secreted and intracellular IL-1RA forms are tissue specific. icIL-1RA is constitutively expressed in tissue sites exposed to environmental factors, such as epithelial cells of the skin, oral cavity, vagina, ovaries and upper respiratory tract [65, 75, 79, 80], while sIL-1RA is found in monocytes, neutrophils and other cells [77].

### Other IL-1 Family Members

The IL-1 family of proteins comprises of several other members in addition to IL-1 (Table 1). IL-18, whose actions are mediated by binding to IL-1R5, is considered an immunomodulatory cytokine important for IFNγ production and is up-regulated by keratinocytes in response to contact sensitizers [81, 82]. IL-33 is a pro-inflammatory cytokine that binds to IL-1R4, but similarly to IL-1α, can also exert its actions directly to the nucleus [83]. IL-36 is an inflammatory protein associated with the development of psoriasis and acts by binding to IL-1R6. Decreased levels of its specific antagonist, IL-36RA, have also been related with the development of pustular psoriasis [84]. Similar to IL-18, IL-37 also binds to and activates IL-1R5. However, unlike IL-18, IL-37 is considered an anti-inflammatory cytokine, where low levels are thought to contribute to disease severity [61]. IL-38 also has predominantly anti-inflammatory actions that are mediated by binding to IL-1R6 [85] where it has been shown to reduce clinical manifestations of systemic lupus erythematosus and arthritis [86].

## IL-1 In Keratinocyte Biology

IL-1α and IL-1RA are constitutively expressed by skin and oral keratinocytes [34, 65, 80, 87]. IL-1β can be found intracellularly [87], but because keratinocytes lack ICE, pIL-1β cannot be cleaved into its 18 kDa mature form and remains inactive and is not reported to be secreted [87, 88]. However, it has been recently reported that normal oral keratinocytes do secrete IL-1β (although in very low levels) that significantly increases with cell aging, although the underlying mechanism is unknown and might be related with the culture conditions (co-culture with irradiated fibroblasts) [65]. To counteract the action of IL-1, keratinocytes mainly express icIL1-RA1 [79, 89], which is localized both in the cytoplasm and inside the nucleus [65], whereas the secreted isoform (sIL-1RA) is either absent or found in very low levels [65, 90]. This makes biological sense as keratinocytes mainly express IL-1α, which is considered an intracellular cytokine, thus an intracellular antagonist is needed to regulate its activity. Although the main actions of icIL1-RA are attributed to its ability to block IL-1R1, icIL1-RA1 is also able to decrease IL-6 and CXCL8 levels by inhibition of the p38 MAPK and NF-κB signaling pathways in an IL-1R1-independent manner [89, 91]. Thus, it is likely that the main icIL-1RA1 functions are related to the regulation of intranuclear IL-1α. Keratinocytes also express IL-1R2 at higher levels than IL-1R1 (resting and when activated). So, both IL-1R2 and icIL-1RA have synergistic roles in regulating IL-1 action on keratinocytes, protecting these cells from excessive autocrine activation of IL-1α [92].

IL-1α and icIL-1RA may have an important role in keratinocyte growth, differentiation, and aging. An *in vitro* study testing the growth conditions for different epithelial cells found that IL-1α inhibited the proliferation of stratified squamous epithelial cells, whereas IL-1RA enhanced it. Moreover, significant growth promotion in normal epidermal keratinocytes was observed upon addition of exogenous IL-1RA [93]. Changes in the ratio of icIL-1RA:IL-1α might help to control growth and differentiation of human skin, as icIL-1RA accumulates in more differentiated cells and IL-1RA expression in oral keratinocytes is positively correlated with the expression of involucrin (a marker of cell terminal differentiation that is restricted to the granular cell layer) [94], whereas IL-1α is uniformly expressed in all keratinocyte maturation stages [33]. icIL-1RA1 is reported to be an important factor in the regulation of oral keratinocyte senescence and the development of the senescence-associated secretory phenotype (SASP) [65]. Cellular senescence corresponds to a cellular state characterized by permanent cell growth arrest in response to different stressors in order to avoid propagation of genetically damaged cells [95]. When cells senesce, they remain metabolically active a develop a SASP characterized by the presence of multiple pro-inflammatory factors which have been related with the development of age-related disorders, including cancer [96]. Oral keratinocytes lacking icIL-1RA1 have been shown to senesce prematurely when compared with keratinocytes expressing icIL-1RA1, and icIL-RA1 was found to regulate the expression of two important SASP factors, IL-6 and CXCL8 [65], which have been associated with the development of malignancies [96].

## IL-1 Signaling In Head and Neck Squamous Cell Carcinoma

IL-1α, IL-1β, and IL-1R1 have been reported to be constitutively expressed in HNSCC [66, 97–99] whilst decreased IL-1RA expression has been observed early in the oral carcinogenesis process [65]. As different members of the IL-1 signaling pathway have reported to have important functions in HNSCC carcinogenesis and tumor progression, we will review them separately.

### IL-1α

IL-1α expression in HNSCC contributes to cell growth and survival and has been considered by some authors as a prognostic factor. In the study of Leon et al. [100], patients with metastatic HNSCC displayed higher expression of IL-1α than patients without metastases. Constitutive IL-1α over-expression was correlated with that of IL-1 family genes, such as IL-1β and IL-1RA. IL-1α expression also correlated with increased cell transmigration of tumor cells across the endothelium, which was inhibited by addition of IL-1RA. IL-1α expression also correlated with different genes that have been associated with metastasis, particularly MMP-9 (a matrix metalloproteinase associated with EMT), PGE_2_ (a product of COX-2 activation associated with metastases of OSCC), VEGF (the most important angiogenic factor in HNSCC) and CXCL8 [100]. The five-year distant metastasis-free survival was 70% for patients with high tumor levels of IL-1α in contrast to 95% for patients with low expression of IL-1α. Patients with increased levels of IL-1α had a 5.3-fold higher risk of developing metastasis and patients with distant metastases had also a significant increase in secreted IL-1α [100].

IL-1α has also been reported to induce the overexpression of IL-6 [97] and CXCL8 in HNSCC cell lines, the latter by inducing NF-κB and AP-1 pathways [36]. This is of importance as IL-6 and CXCL8 are considered important “oncogenic cytokines”, as they are able to cause EMT [101], stimulate angiogenesis and tumor growth [102, 103], disrupt cell-cell communication, impede macrophage function and promote epithelial and endothelial cell migration and invasion [104]. NF-κB is considered a key factor in the regulation of the inflammatory infiltrate observed in the TME [105] and has been associated with the acquisition of a malignant phenotype of HNSCC, as is associated with tumor angiogenesis [106], EMT [107], invasion [108, 109] and metastasis [110]. In addition, inactivation of NF-κB in HNSCC suppressed cell survival and expression of IL-1α, IL-6, CXCL8 and GM-CSF in a murine model of head and neck cancer [111] and its aberrant expression is associated with poor prognosis in solid cancers [112]. AP-1 expression increases with HNSCC progression and induces bcl-2 expression that is associated with suppression of apoptosis and resistance to chemoradiation therapy [113] (**Figure 3**).

### IL-1β

The mechanism by which IL-1β is constitutively overexpressed in HNSCC is not clear, but a single nucleotide polymorphism of the IL-1β gene could explain this. In fact, IL-1β-511 polymorphism has been reported to be a significant risk factor for the development of OSCC [114]. IL-1β is identified as a key node gene in the tumor microenvironment (TME) of OSCC *in vivo* [115]. Keratinocytes lack ICE and therefore should not be able to produce the mature active form of IL-1β. However, other proteases are able to cleave IL-1β precursor form, suggesting that pIL-1β can be processed after secretion by other proteases that are present in the TME [116]. In agreement with this, IL-1β produced by oral keratinocytes and HNSCC cells is biologically active. Interaction of IL-1β with the TME leads to monocyte recruitment that then differentiate into tumor-associated macrophages (TAMs) whose increased levels in HNSCC are associated with poor prognosis [12, 117]. Also, stimulates the production of numerous cytokines by different cell types, such [116] as cancer-associated fibroblasts (CAFs), normal fibroblast, endothelial cells, neutrophils as well as oral dysplastic and cancer cells, among others [10, 11, 98, 118], through an IL-1-dependent innate immune response.

**Figure 1:**
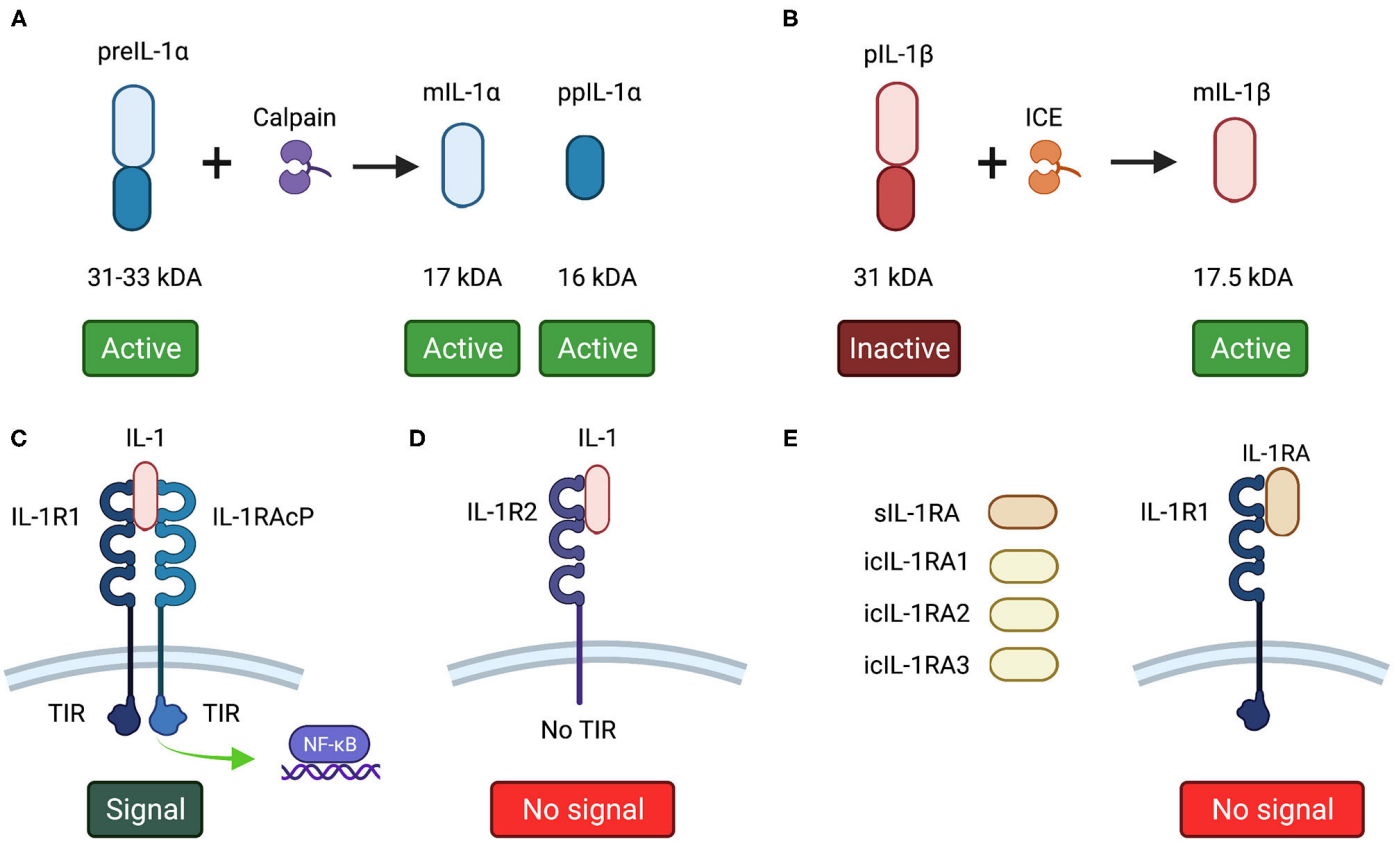
**(A)** IL-1α and **(B)** IL-1β forms. **(C)** IL-1R1 agonist receptor. To be active, IL-1R1 needs the binding of IL-1RAcP. Both IL-1R1 and IL-1RAcP have a TIR domain, which after a series of phosphorylation following IL-1 binding, activate NF-kB. **(D)** IL-1R2 decoy receptor binds to IL-1 without triggering any agonist action as lacks of a TIR domain. **(E)** IL-1RA variants bind to IL-1R1, blocking the binding of IL-1 without recruiting IL-1RAcP, thus triggers no agonist action. This image was created with Biorender.

**Figure 2:**
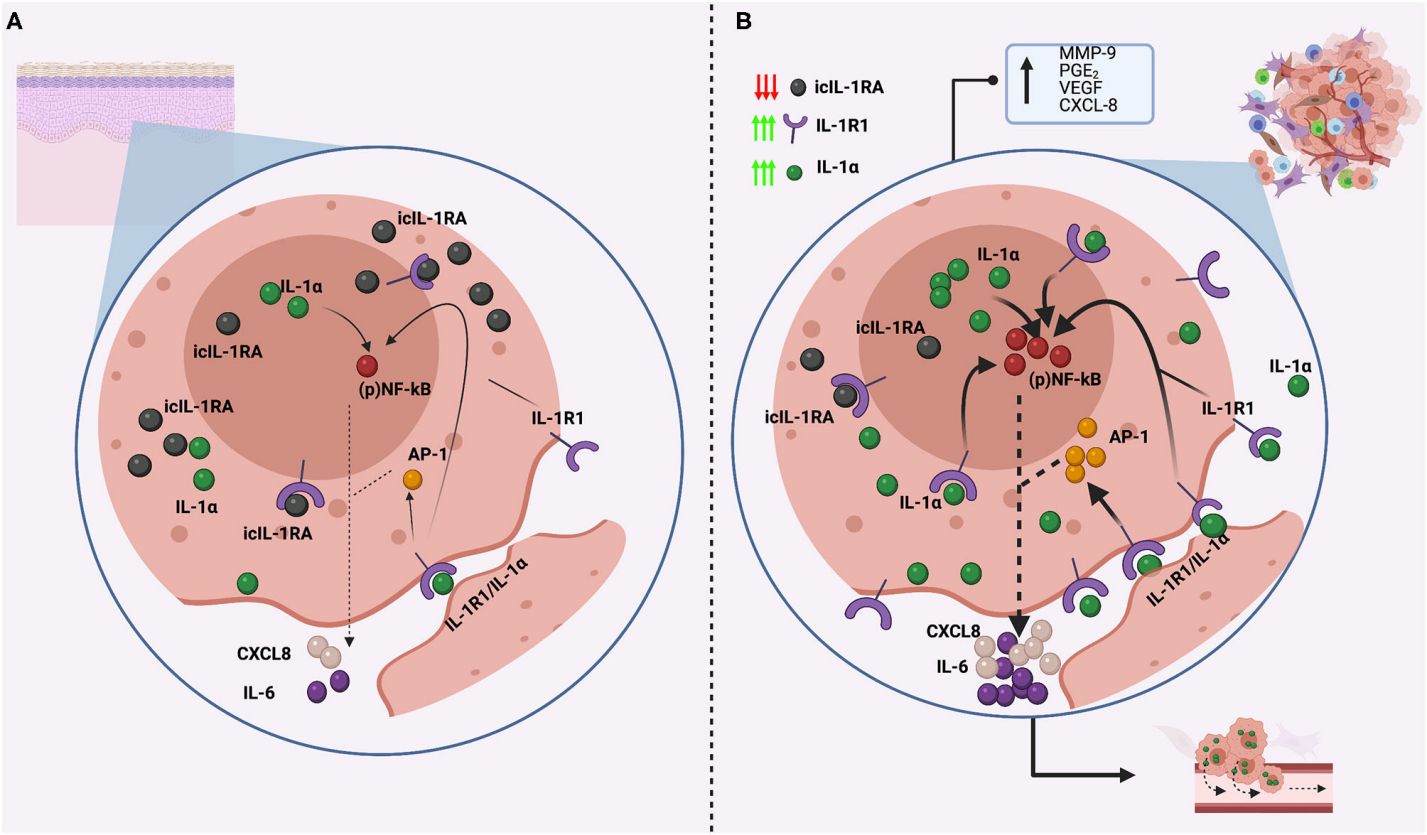
Comparison of IL-1α expression and regulation in normal head and neck keratinocytes **(A)** and in cancerous head and neck keratinocytes **(B)**. **(A)** Normal keratinocytes express low levels of IL-1α, which is not normally secreted, and is regulated by icIL1RA, which is expressed in abundancy to efficiently counteract IL-1α actions. This balance secure low NF-kB activity with low production of other inflammatory molecules, such as IL-6 and CXCL-8. **(B)** In cancerous head and neck keratinocytes, icIL-1RA expression is downregulated, whereas IL-1R1 and IL-1α are constitutively upregulated, generating an imbalance in IL-1 regulation. IL-1α can localize intracellularly and interact directly to the nucleus, can be attached to the cellular membrane (membrane IL-1α) or can be released to the extracellular space. Membrane and secreted IL-1α can both bind to IL-1R1 receptors and activate NF- κB and AP-1 transduction pathways, resulting in the release of IL-6 and CXCL8, which are considered oncogenic cytokines as are associated with tumor growth and metastasis. Also, intranuclear IL-1α can interact directly with the nucleus inducing NF-κB activation in a non-IL-1R1 dependent manner. This also results in the release of IL-6 and CXCL8, which are both overexpressed in HNSCC. This image was created with Biorender.

The IL-1β found in the TME is also produced by other cells in addition to HNSCC cells. A recent report demonstrated that IL-1β produced by CAFs induces CCL22 mRNA overexpression in oral cancer cells. CCL22 is implicated in the recruitment of T regulatory cells, and its expression in oral cancer patients has been associated with a reduced disease-free survival [119]. Tumor-associated macrophages (TAMs) also secrete IL-1β, which, together with the actions of TNF-α stimulate tumor angiogenesis by inducing the release of VEGF and CXCL8 by HNSCC cells [120]. This creates an inflammatory TME that can predispose to tumor progression [121] (Figure 1). For example, in HNSCC IL-1 signaling drives neutrophil and monocyte recruitment [10], and accumulation of these tumor-associated leukocytes has been associated with poor prognosis [13, 122]. The IL-1/IL-1R axis mediates chemokine release from normal tonsillar fibroblasts (NTF) induced by HPV-negative oropharyngeal carcinoma (OPC) cells, which can be reverted with IL-1 inhibition [11]. This is of significance, as for example, CXCL1 and MMP-1 produced by CAFs in response to IL-1β from OSCC cells, increased the invasion and migration capabilities of OSCC cells [123]. Also, IL-1 released from HNSCC cells has been shown to stimulate COX-2 production by CAFs [124] that correlated with lymphangiogenesis [125] and E-cadherin regulation, important factors for epithelial-to-mesenchymal transition (EMT) development [126], and increased risk of distant metastases [127]. In OSCC, IL-1β produced by tumor cells can act in a paracrine manner, inducing the expression of fascine that is associated with ECM degradation and tumor cell invasion [128] (Figure 2).

The oncogenic properties of IL-1β have also been demonstrated using *in vivo* models. In a mouse oral cancer model in which carcinogenesis was induced by mimicking tobacco and areca nut carcinogens, an increase in pIL-1β mRNA positively correlated with the presence of malignant change (from normal, to mild, through severe dysplasia to OSCC). In agreement with these findings, OSCC and dysplastic cell lines from smokers and/or betel quid chewers had higher IL-1β levels than controls, with inflammasome components constitutively expressed in OSCC cells that allows the cleavage of pIL-1β into mIL-1β. IL-1β might have an important role in the induction of EMT in OSCC, as in the same study, OSCC cells treated with IL-1β showed upregulation of Snail and Slug (two repressors of E-cadherin expression), increased vimentin expression, and downregulation of E-cadherin. This was also correlated with a change in cell morphology, from a squamous cell-like shape, in cells not exposed to IL-1β, to a spindle-like shape in OSCC cells exposed to IL-1β. The migration capacity of OSCC treated with IL-1β increased significantly after 48 hours, compared to untreated OSCC. These findings strongly suggest a role of IL-1β in EMT in OSCC [98], which has also been reported by others [126, 129, 130]. This is also supported by the fact that IL-1β silencing reduces OSCC tumor size *in vivo* [115] and that elevated IL-1β expression has been related with lymph node metastasis of OSCC [131].

### IL-1RA

Several gene expression profiles of HNSCC have shown that *IL-1RN* is downregulated in HNSCC when compared to matched normal oral mucosa [132–139]. In addition, *IL-1RN* was reported to be a reliable marker when predicting the presence or absence of HNSCC in tissue samples in a cohort of 46 patients with HNSCC, with sensitivity and specificity of 93.5 and 95.7%, respectively [138]. Despite this, very little is known about the role of IL-1RA in HNSCC.

**Figure 3:**
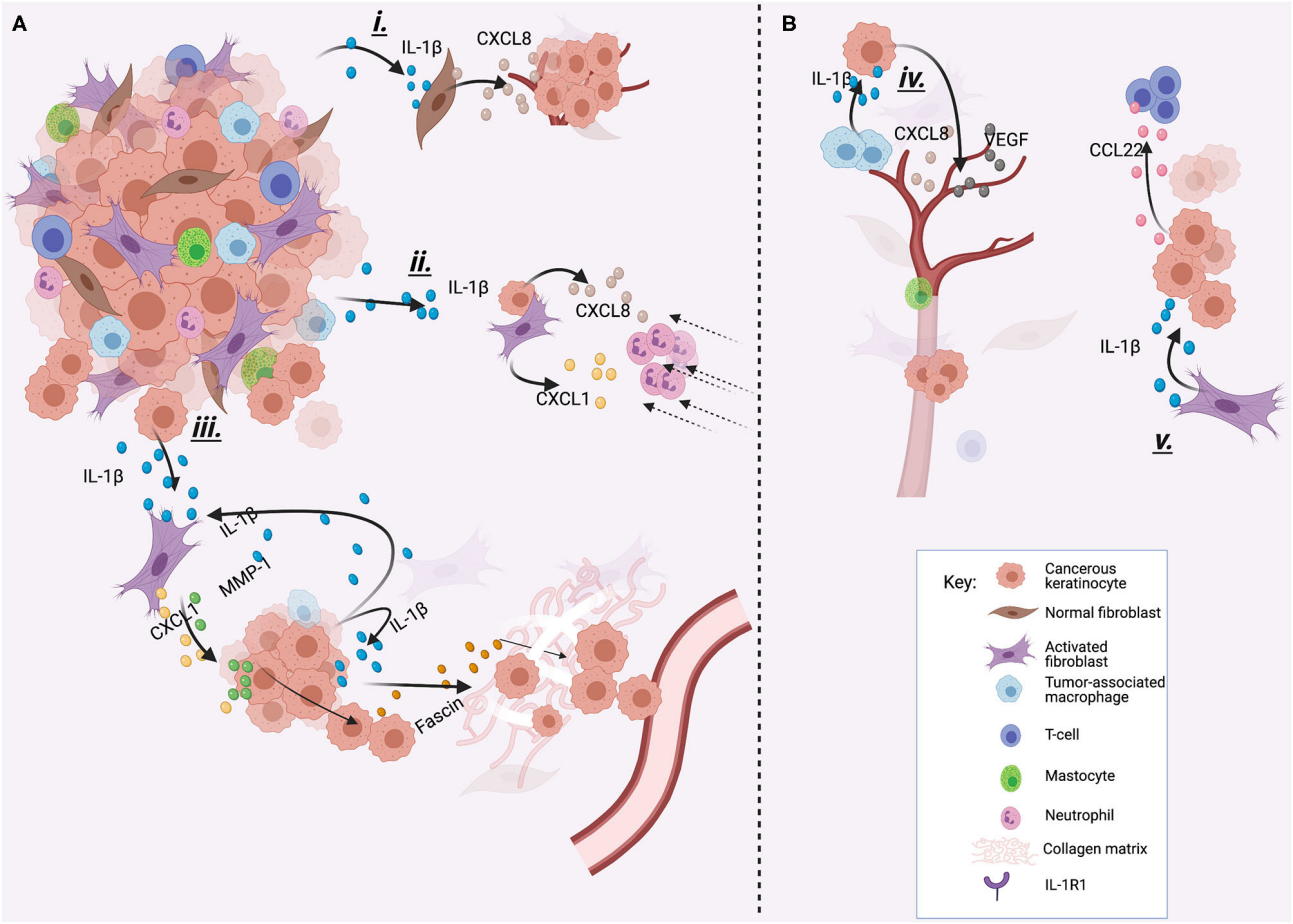
Different examples of how IL-1βcan promote cancer progression. IL-1β can be produced by tumor cells **(A)** or by other cells of the TME **(B)**. (i) IL-1β produced by tumor cells stimulates CXCL8 release from normal oral fibroblasts, which increases tumor growth. (ii) IL-1β stimulates CXCL1 and CXCL8 production by CAFs and tumor cells which attracts neutrophils to the TME, and neutrophil accumulation has been related with poor outcome in HNSCC. (iii) IL-1β released by tumor cells induces CXCL-1 and MMP-1 secretion by CAFs. This induces invasion and migration of cancer cells. Also, secreted IL-1β can act in a paracrine way inducing fascine release, which helps in the degradation of the extracellular matrix and invasion. (iv) TAMs produce IL-1β to stimulate cancerous cells to produce VEGF and CXCL8, inducing angiogenesis. (v) IL-1β produced by CAFs induces the release of CCL22 by tumor cells, which recruits T regulatory cells, who have been associated with worse prognosis. This image was created with Biorender.

Von Biberstein et al. [99] reported an imbalance in the IL-1:IL-1RA ratio in HNSCC when compared to healthy patients, that was attributed mainly to an increase in the levels of IL-1α and IL-β, but also to a decrease in the levels of IL-1RA. These authors also reported higher expression of IL-1RA in the more differentiated epithelial cells within the tumors. IL-1RA protein expression decreases progressively during oral carcinogenesis and in HNSCC [65], which is in agreement with a previous report [80] and IL-1R2, the other IL-1 inhibitor, is not able to compensate for IL-1RA lack of expression [65]. Also, IL-1RA levels decrease significantly in immortal normal and dysplastic oral keratinocytes when compared to their mortal counterparts. This suggest that IL-1RA downregulation during the carcinogenic process might be an important step for the acquisition of a malignant phenotype, primarily because the binding of IL-1 to IL-1R1 is not inhibited, allowing dysregulated activation of the IL-1/IL-1R1 axis that can predispose to the carcinogesis process in different ways (Figures 1, 2). Moreover, In oral keratinocytes, icIL-1RA1 regulates IL-6 and CXCL8 secretion, most likely by interfering with NF-κB activation [65]. Both IL-6 and CXCL-8 secretion is canonically regulated by NF-κB, and icIL-1RA1 is able to regulate NF-κB activation wheter by inhibiting IL-1 binding to IL-1R1, or by directly interfering with the NF-kB signaling pathway (see below). Thus, a downregulation of IL-1RA would allow overexpression of these cytokines (Figure 3).

There has been much debate about the specific functions of icIL-1RA. icIL-1RA is able to bind to IL-1R1, but as a stricktly intracellular molecule, it is more likely that the main functions of icIL-1RA are not related to IL-1R1 binding. IL-1R1 is located on the membrane surface, thus, icIL-1RA would require secretion into the extracellular space for it to block IL-1R1, although IL-1R1 intranuclear localization in oral dysplastic and cancer cells has been recently described [65]. It has been proposed that the main function of icIL-1RA is to counteract the intranuclear action of IL-1α. icIL-1RA may act intracellularly by binding to other cytoplasmic proteins in order to interfere with the downstream cascade. In fact, icIL-1RA has been reported to interact with the third component of the COP9 signalosome (CSN3) inhibiting CSN-associated kinases [91]. The signalosome (CSN) is found in the cytoplasm and nucleus of all mammalian cells, and among other functions, it has kinase activity that induce phosphorylation of proteins involved in signal transduction. When interacting with CSN3, icIL-1RA1 inhibits phosphorylation of p53, c-Jun and IκB thereby inhibiting IL-1α-mediated IL-6 and CXCL8 transcription. These inhibitory actions may also affect the p38 MAPK signal transduction pathway, as transfected keratinocytes with icIL-1RA1 showed no detectable phosphorylated p38 MAPK when stimulated with IL-1α [91]. Similar icIL-1RA1 inhibitory mechanisms have also been reported in intestinal epithelial cells [89]. Böcker et al. [140] also reported inhibition of IL-1-induced CXCL8 expression by icIL-1RA1, but did not specify the mechanism underlying this inhibition. It has been proposed that the role of CSN3 is to bring icIL-1RA1 close to a kinase in order to inhibit its action. So, icIL-1RA1 would block an upstream kinase in the p38 MAPK or NF-κB pathways, indirectly inhibiting p38 MAPK or NF-κB phosphorylation and consequently, its downstream products, such as IL-6 and CXCL8 [91].

It can be hypothesized then, that downregulation of icIL-1RA in HNSCC could lead to de-regulated expression of pro-inflammatory cytokines related to cancer development by allowing the un-controlled activation of IL-1α and NF-κB (Figure 3). This speculation is based on the finding that OSCC constitutively express higher levels of IL-1α and NF-κB than healthy controls [8, 100, 118, 141] and that levels of IL-6 and CXCL8 are elevated in OSCC [142, 143]. In addition, IL-1α can interact directly with nuclear DNA an induce malignant transformation [40]. Thus, if endogenous levels of IL-1α are constitutively overexpressed, and its mains inhibitor (icIL-1RA) is downregulated, there could be more chances for icIL-1α to induce malignant transformation. In agreement with this hypothesis, a recent study comparing nuclear and cytoplasmic IL-1α expression in OSCC showed that the high expression of nuclear IL-1α in combination with EGFR, was associated with perineural invasion and high risk of recurrence and worse progression-free survival, compared to OSCC expressing none or moderate nuclear IL-1α in combination with EGFR [144]. These studies suggests that uncontrolled nuclear IL-1α activity might be of clinical importance.

There is some controversy about the role of IL-1RA in HNSCC. Shiiba et al. [80] reported an increase of IL-1RA (T3/T4) compared to early OSCC cases (T1/T2), suggesting that IL-1RA expression could increase tumor progression. Similar observations have been reported by other authors in gastric [145] and cervical carcinomas [146], reporting a more aggressive behavior from IL-1RA expressing tumors. A possible explanation to this could be that over time, endogenous IL-1 antagonism (which is likely to be beneficial in antagonizing disease progression) changes the tumor phenotype to one that is less susceptible to IL-1 inhibition. Thus, the disease progresses, and IL-1RA levels remain high [71]. Also, most of the aforementioned studies measured sIL-1RA, so the increase in sIL-1RA may be due to a decrease in icIL-1RA levels. Nevertheless, these contradictory functions of IL-1RA in different cancers only highlights the multiple functions that IL-1RA displays in relation to the specific tissue, cell type or the microenvironment in which it is present.

### IL-1R1

A number of different polymorphisms of the *IL1R1* gene have been related with a reduced (rs956730) or increased (rs3917225) risk for developing HNSCC [147]. IL-1R1 is overexpressed by oral dysplastic and OSCC cells compared to normal oral keratinocytes [65], which seems to provide phenotypic advantages. IL-1R1 promotes oral cancer growth and metastasis by upregulating CXCR4 (a chemokine receptor involved in tumor progression, angiogenesis and metastasis) after IL-1β stimulation, and IL-1R1 inhibition with recombinant IL-1RA has shown to reverse these effects [66]. IL-1R1 is also constitutively expressed by normal oral fibroblasts [11]. This is of importance as HNSCC cells are able to secrete IL-1β which stimulates other cells of the TME (such as fibroblasts) to generate chemokines and other inflammatory molecules creating an inflammatory TME with cancer promoting properties [11].

## Translational Potential

### Utility as a Saliva Biomarker

The discovery that saliva contains molecules that are able to translate the presence or activity of local or systemic diseases has opened a new diagnostic field known as salivary diagnostics [148]. The use of saliva as a diagnostic method is very practical, as saliva can be collected in an easy, non-invasive way. As there is evidence suggesting that IL-1α, IL-1β, and IL-1RA are involved in the pathogenesis of HNSCC and can be detected in the saliva of cancer patients [149–151], different studies have explored their possible use as diagnostic or prognostic biomarkers for this cancer.

IL-1β is overexpressed in the saliva of oral cancer patients compared to oral leukoplakia and control patients [152, 153] and IL-1β salivary levels have been shown to discriminate between OSCC subjects and controls [154–156], but not between oral potentially malignant disorder (OPMD) patients and healthy subjects [155]. The reported AUC of salivary IL-1β to differentiate between OSCC and control individuals varies between 0.729 and 0.7724 [154, 155] but increases to 0.901 when considering only late stage OSCC [155]. Also, the discriminatory power of salivary IL-1β increases when used with other markers, such as CXCL8, SAT1 and DUSP1 [156]. In the study by Singh et al. salivary IL-1β failed to distinguish between post-treatment OSCC individuals and healthy subjects, suggesting a normalization of IL-1β salivary levels after tumor removal [155]. In agreement, a study in which the authors analyzed the expression of 50 cytokines (including IL-1β, IL-1 α, and IL-1RA) in the saliva of 16 OSCC patients before and after surgical intervention, showed a significant decrease in salivary IL-1β levels after tumor resection. No significant changes in other cytokine levels were reported [150]. Similar results were also reported elsewhere [157]. IL-1α expression is also reported to be increased in the saliva of tongue SCC (TSCC) patients compared to controls and has also been associated with tumor growth pattern [9, 158]. Patients presenting with endophytic TSCC exhibited significantly higher IL-1α levels compared to exophytic TSCC, which correlated with a decreased survival rate in the group of endophytic tumors [158].

IL-1RA can also be detected in the saliva and its expression is reported to be significantly decreased in the saliva of OPMD and OSCC patients compared to healthy controls [151]. Opposite to salivary IL-1RA, plasma circulating IL-1RA levels have been shown to be increased and OSCC patients and correlated with tumor size, but were not related with different outcome measures [159]. By itself, salivary IL-1RA displayed a poor performance in diagnosing OSCC, but in combination with other proteins (SLC3A2 and S100A2), it was able to distinguish between individuals with OSCC from healthy controls and OPMD patients, with AUC of 0.89 and 0.87 respectively [151].

The use of salivary IL-1α, IL-1β, and IL-1RA as HNSCC biomarkers is promising, but as there are many local and systemic diseases that can give rise to elevated salivary IL-1 or decreased IL-1RA levels (e.g., periodontal disease, oral lichen planus, Sögren's syndrome) [94, 160, 161], which increases the likelihood of false positives, more clinical studies are needed before translating this into clinical practice.

### Therapeutics

Many beneficial functions of IL-1 inhibition have been described in different cancers, thus, targeting IL-1 has been proposed as a possible therapy for IL-1 expressing tumors, such as melanoma, gastric and breast cancers, among others [162]. In gastric cancer, recombinant IL-1RA inhibited tubule formation [163] and reduced proliferation and migration of endothelial cells *in vitro* in a dose-dependent manner [164]. Similar results have been reported in breast cancer. A murine experimental breast cancer model showed that treatment with anakinra (a recombinant form of IL-1RA with FDA approval for the treatment of rheumatoid arthritis and cryopyrin-associated periodic syndromes) reduced the size and number of bone metastases as well as tumor angiogenesis [165]. Taken together, these data suggest that rIL-1RA could be a beneficial alternative for the inhibition of tumor-dependent angiogenesis, probably by reducing the production of VEGF, CXCL8, endothelin-1, IL-1β and hepatocyte growth factor (HGF) [164–166].

Anakinra has been used in clinical trials for the treatment of some cancers. The first study to use anakinra as a cancer treatment was a phase II clinical trial of pre-multiple myeloma. Anakinra in combination with dexamethasone was found to increase the progression-free survival as well as overall survival in patients at high risk of progressing to multiple myeloma, by targeting the IL-1/IL-6 pathway [167]. In refractory metastatic colorectal cancer, anakinra in combination with fluorouracil (an anti-metabolite) and bevacizumab (anti-EGF monoclonal antibody) showed good efficacy with low toxicity. Currently, there are several clinical trials where recombinant IL-1RA is being tested either as a monotherapy or in combination for the treatment of different cancers, including multiple myeloma, prostate, breast, pancreatic, and colorectal cancers (https://clinicaltrials.gov/ct2/results?cond=Cancer&term=termIL1RA+OR+Anakinra&cntry=&state=&city=&dist=). Nevertheless, care must be taken when considering IL-1RA therapy for HNSCC treatment, as recombinant IL-1RA is likely reduce the innate immunity response in already ill patients, which in theory, could worsens the disease. Thus, IL-1RA replacement therapy may be only appropriate for IL-1 producing tumors [162].

Exogenous IL-1RA (i.e., anakinra) corresponds to the secreted isoform of IL-1RA, which is present in very low levels in oral keratinocytes, as oral keratinocytes constitutively express icIL-1RA1. It is not entirely clear how exogenous IL-1RA works. It is thought that exogenous IL-1RA acts in a similar manner to sIL-1RA, by blocking IL-1R1 on the cell membrane. Nevertheless, exogenous IL-1RA has been shown to be incorporated into the cytoplasm of cardiac myocytes during ischemia, mimicking the intracellular form of IL-1RA, at least in terms of intracellular distribution [168]. Whether exogenous IL-1RA can replace icIL-1RA (which expression is lost during the malignant transformation process of oral keratinocytes and OSCC) functions is not known.

Despite the data supporting an oncogenic role of the IL-1/IL-1R1 axis in HNSCC, there is a lack of studies that have explored the use of IL-1 inhibition for HNSCC treatment. A recent study [115] showed that exogenous IL-1RA can inhibit the growth of Cal27 cells (a tongue squamous adenocarcinoma cell line) *in vitro*, but more importantly, can potentially interrupt the oral carcinogenesis process *in vivo*. Submucosal injections of IL-1RA into the tongue of mice during 4NQO-induced oral carcinogenesis interrupted the malignant transformation process. This was done presumably by downregulating genes that were upregulated during the 4NQO-induced carcinogenesis process, such as the oncogene Myc and COX-2 [115]. In addition, anakinra has been shown to overcome erlotinib (an EGFR inhibitor) resistance in a HNSCC mouse xenograft cancer model, suggesting its use as a possible strategy to overcome EGFR inhibitor resistance for HNSCC treatment [169]. Although these initial data are promising, there are no clinical trials that have assessed anakinra for HNSCC treatment and more research in this area is warranted.

## Conclusion

There is compelling evidence that the IL-1 signaling pathway is deregulated in HNSCC, with overexpression of agonistic molecules and downregulation of inhibitory factors. This results in a dysregulated signaling pathway that mediates the development of a pro-inflammatory microenvironment prone to tumor development, progression and metastasis. Oral carcinogenesis is a multistep process which includes three phases: initiation, promotion, and progression. The available evidence suggests IL-1 signaling to influence the promotion and progression phases of the malignant transformation process. The increased presence of IL-1 in HNSCC support the idea that salivary IL-1 could be of use as a screening tool for the early detection of cancer, probably as part of a biomarker panel rather than as a single marker. Early data shows promise, although more rigorous studies are needed before this can be translated into clinical practice. IL-1 inhibition is already being tested as a possible treatment alternative for different cancers, such as myeloma, breast, pancreatic and colorectal cancers, and there are *in vivo* animal studies showing promising results for HNSCC treatment. However, there is still a long way to go before this can be applied in a clinical setting.

## Author Contributions

All three authors contributed equally to the writing of this review. The concept and outline were agreed upon by all. SN wrote the initial draft and constructed the figures. All authors agree on the final version for submission.

## Conflict of Interest

The authors declare that the research was conducted in the absence of any commercial or financial relationships that could be construed as a potential conflict of interest.

## Publisher's Note

All claims expressed in this article are solely those of the authors and do not necessarily represent those of their affiliated organizations, or those of the publisher, the editors and the reviewers. Any product that may be evaluated in this article, or claim that may be made by its manufacturer, is not guaranteed or endorsed by the publisher.
